# Feasibility of extreme dose escalation for glioblastoma multiforme using 4π radiotherapy

**DOI:** 10.1186/s13014-014-0239-x

**Published:** 2014-11-07

**Authors:** Dan Nguyen, Jean-Claude M Rwigema, Victoria Y Yu, Tania Kaprealian, Patrick Kupelian, Michael Selch, Percy Lee, Daniel A Low, Ke Sheng

**Affiliations:** Department of Radiation Oncology, University of California, Los Angeles, 200 Medical Plaza Way, Suite B265, Los Angeles, USA

## Abstract

**Background:**

Glioblastoma multiforme (GBM) frequently recurs at the same location after radiotherapy. Further dose escalation using conventional methods is limited by normal tissue tolerance. 4π non-coplanar radiotherapy has recently emerged as a new potential method to deliver highly conformal radiation dose using the C-arm linacs. We aim to study the feasibility of very substantial GBM dose escalation while maintaining normal tissue tolerance using 4π.

**Methods:**

11 GBM patients previously treated with volumetric modulated arc therapy (VMAT/RapidArc) on the NovalisTx™ platform to a prescription dose of either 59.4 Gy or 60 Gy were included. All patients were replanned with 30 non-coplanar beams using a 4π radiotherapy platform, which inverse optimizes both beam angles and fluence maps. Four different prescriptions were used including original prescription dose and PTV (4πPTV_PD_), 100 Gy to the PTV and GTV (4πPTV_100Gy_), 100 Gy to the GTV only while maintaining prescription dose to the rest of the PTV (4πGTV_100Gy_), and a 5 mm margin expansion plan (4πPTV_PD+5mm_). OARs included in the study are the normal brain (brain – PTV), brainstem, chiasm, spinal cord, eyes, lenses, optical nerves, and cochleae.

**Results:**

The 4π plans resulted in superior dose gradient indices, as indicated by >20% reduction in the R50, compared to the clinical plans. Among all of the 4π cases, when compared to the clinical plans, the maximum and mean doses were significantly reduced (p < 0.05) by a range of 47.01-98.82% and 51.87-99.47%, respectively, or unchanged (p > 0.05) for all of the non-brain OARs. Both the 4πPTV_PD_ and 4π GTV_100GY_plans reduced the mean normal brain mean doses.

**Conclusions:**

4π non-coplanar radiotherapy substantially increases the dose gradient outside of the PTV and better spares critical organs. Dose escalation to 100 Gy to the GTV or additional margin expansion while meeting clinical critical organ dose constraints is feasible. 100 Gy to the PTV result in higher normal brain doses but may be tolerated when delivered in proportionally increased treatment fractions. Therefore, 4π non-coplanar radiotherapy on C-arm gantry may provide an accessible tool to improve the outcome of GBM radiotherapy through extreme dose escalation.

## Introduction

Glioblastoma multiforme (GBM) is a devastating disease with a dismal survival rate. Even with aggressive surgical and chemoradiation, the average survival period is 12–14 months after diagnosis [1]. Although radiotherapy has been shown to delay recurrence and prolong patient survival, GBM is remarkably resistant to treatment and has a high recurrence rate that contributes to patient mortality. The recurrence overwhelmingly occurs in or near the high-dose radiation field and original tumor site [2]. The biology behind the radiation resistance is not well understood but the pattern of recurrence suggests that there may be surviving tumor cells within or near the high dose area. This observation has motivated dose escalation studies. An increased survival period with dose escalation up to 60 Gy has been observed based on a non-randomized clinical trial [3], although this improvement was achieved without advancing the local control rates.

Dose escalation studies utilizing three-dimensional conformal radiotherapy showed that, even with dose escalation to 70 Gy and 80 Gy, the recurrences from highly conformal treatments are still predominantly local [4,5]. Further dose escalation to 90 Gy did not improve local control, instead, worsened the patient survival period possibly due to the increased treatment related toxicity [4,5]. Studies that have attempted other methods to treat GBM, such as a phase II trial utilizing weekly stereotactic radiotherapy boost [6] and a phase I trial of hypofractionated IMRT with temozolomide chemotherapy [7], have also found predominantly local recurrence and no survival benefit.

Further dose escalation using external X-ray beams alone was deemed infeasible due to normal tissue dose constraints. To increase the tumor dose, a phase II prospective trial using combined proton and photon therapy to deliver 90 Gy showed improved central tumor control, and increased median survival period to 20 months [8]. Although the total radiation dose is the same as the photon trial, the treatment was delivered in a bis in die (b.i.d. or twice a day) fashion, resulting in a substantially greater biological equivalent dose. Alternatively, a Northern California Oncology Group (NCOG) trial utilized brachytherapy to boost the tumor dose [9,10]. After being treated to a median prescription dose of 59.5 Gy via external beam radiation therapy, patients received a boost dose to the GTV from an ^125^I (10–40 mCi) with a dose rate of 40–60 cGy/hr to a median boost dose of 50.88 Gy within 5–6 days, totaling to approximately 110 Gy. The results from this non-randomized clinical trial showed that the dose escalated GBM patients achieved an improved 1 and 2 year survival rate of 87% and 57% when compared to the control group receiving external beam radiation only of 40% and 12.5%, respectively. In a recurrent GBM trial using the same regimen, 10% (3) patients achieved a 5 year disease-free survival although the overall pattern of recurrence after radiotherapy and chemotherapy remained predominantly local [11]. While this treatment regimen is toxic and often results in steroid dependence and surgical removal of the necrosis induced by the aggressive treatment, it indicated the potential for substantially improving GBM patient treatment outcomes with very aggressive dose escalation to 100 Gy or greater.

Brain brachytherapy implant is associated with significant risk and the procedure itself may have contributed to the severe side effects. The complexities involved in the brachytherapy procedures and resulting patient management have prevented it from being widely and sustainably applied. At the times of the aforementioned clinical trials, external beam alone was deemed insufficiently conformal to deliver such high doses due to normal tissue dose constraints. Brain necrosis is the dominant presentation of treatment related toxicity from dose escalation studies beyond 60 Gy. Therefore, reducing the high dose spillage by increasing dose distribution compactness is critical to the success of dose escalation.

With the recent advent of highly conformal non-coplanar intensity modulated 4π therapy, markedly improved radiation dose conformity has been demonstrated [12-14]. Specifically, a significant reduction of the high dose spillage has been observed. This dosimetric improvement may afford GBM dose escalation to 100 Gy or greater using external beam only with the hope of achieving a survival benefit, without significantly increasing toxicity.

## Patients & methods

11 GBM Patients, 8 male and 3 female, were included in the study. The maximum, minimum, and median ages were 66 years, 26 years, and 49 years, respectively. Detailed patient information of 11 GBM Patients included in the study is shown in Table 1. The gross tumor volume (GTV) was delineated on a contrast enhanced magnetic resonance imaging (MRI) manually fused to the CT simulation scan. The GTV was defined as the gross disease enhancing on the T1-weighted MRI images plus any abnormal enhancement on T2-weighted and FLAIR images. The clinical target volume (CTV) was then defined as the GTV + 2 cm expansion with anatomical considerations. Lastly, the planning target volume (PTV) was defined as the CTV + 0.5 cm expansion. Patients were treated to a prescription dose of either 59.4 Gy or 60 Gy to 95% of the PTV. For all patients, highly conformal volumetric modulated arc therapy (VMAT) plans were designed using Eclipse (Varian Medical Systems, Palo Alto, California) with 2 to 4 coplanar or non-coplanar arcs as necessary to achieve optimal critical structure sparing. Final dose calculation was performed using the analytical anisotropic algorithm (AAA) planning algorithm. This planning method was shown equivalent or superior to static beam IMRT, particularly with the additional partial arcs [15]. The organs-at-risk (OARs) typically involve the optical apparatus and brainstem, which are subject to maximal dose constraints and the brain, which has tolerances that depend on both the maximal and volume dose. OAR dose tolerances were defined according to RTOG protocol 0825 with the following maximum point dose constraints: chiasm <56 Gy, lens <7 Gy, brainstem <60 Gy, optic nerve <55 Gy and cochlea <45 Gy. It has been reported that in conventionally fractionated radiotherapy, a maximal dose of 72 Gy (range 60 – 84Gy) results in a 5% risk of symptomatic radiation necrosis at 5 years and the risk increases to 10% with 90 Gy [16]. In this study, normal brain doses greater than 60 Gy as well as the mean brain doses were constrained. In addition, it had been observed in brain SRS studies that there is a rapid rise of brain necrosis when the volume of brain receiving 12 Gy or greater (V12) is more than 5–10 cm^3^ [17]. This single fraction dose is biologically equivalent to 36 Gy delivered in 2 Gy daily fractions. Therefore, additional DVH points were used to panelize normal brain receiving 30–36 Gy in the VMAT plans. Treatment was planned for a NovalisTx system equipped with a 2.5 mm leaf-width MLC.

**Table 1:** **Basic patient information**

**Patient ID**	**PTV vol(cc)**	**5 mm PTV vol(cc)**	**GTV vol(cc)**	**Brain vol(cc)**	**Non-tumor brain vol(cc)**	**Prescription Dose (Gy)**	**Number of arcs used**	**Site**
1	262.49	379.9	66.7	1724.3	1492.0	60	**2 non-cop**	L frontal
2	265.78	400.2	71.6	1406.6	1171.2	60	**2 non-cop**	L temporal
3	231.97	368.2	56.7	1163.8	949.6	59.4	**3 non-cop**	R temporal
4	252.33	363.5	67.9	1539.2	1321.2	59.4	**3 non-cop**	L temporal
5	272.41	416.3	101.8	1356.1	1123.9	59.4	**3 non-cop**	L frontal
6	426.32	609.5	132.2	1418.1	1041.6	59.4	**3 non-cop**	R parietal
7	429.82	548.5	124.6	1241.9	890.7	59.4	**2 coplanar**	L frontal
8	377.77	555.0	120.0	1473.9	1102.0	59.4	**2 coplanar**	L frontal
9	402.40	567.6	144.5	1657.3	1306.7	59.4	**2 coplanar**	L frontal
10	196.42	342.2	39.8	1542.5	1350.6	59.4	**2 non-cop**	R Occipital
11	463.52	699.0	143.5	1398.6	1004.6	59.4	**4 non-cop**	Bilateral occpt-temp

4π non-coplanar radiotherapy was implemented following previous publications [12-14]. Briefly, 4π radiotherapy is a non-coplanar planning platform established on existing C-arm gantry linacs. The 4π optimization method starts with a candidate pool of 1162 beams evenly distributed throughout the 4π solid angle space with 6° of separation between adjacent beams. Beams causing collision between the gantry and couch or patient are excluded using a collision map based on the 3D surface image of the machine and a human subject. The remaining pool of candidate beams subdivided into 5 × 5 mm^2^ beamlets, are calculated utilizing convolution/superposition of Monte Carlo calculated 6 MV poly-energetic kernels. A column generation algorithm [18] is used to iteratively select and optimize beam fluence until the desired number of beams is reached. In this case, 30 non-coplanar beams were utilized for each patient. Dose constraints similar to those of VMAT plans were applied.

The dose calculation resolution was 2.5 mm on both the clinical and 4π plans, which were then exported to a Matlab program (Computational Environment for Radiation Research CERR, Washington University) for dosimetric comparison.

Quantitative analysis was used to compare the mean and maximum OARs and PTV doses, as well as R50, a measurement of high dose spillage or dose gradient outside the PTV, which is defined as the ratio between 50% isodose volume and the PTV. V30 and V36, markers for potential brain necrosis, were assessed. To test how dose escalation affects the low dose region, V5, V10, and V20 were also evaluated. “Vx” is defined as the volume that has “x Gy” of dose or higher.

The 4π plans with the original prescription doses (4πPTV_PD_) were compared to the clinical plans. To test the hypothesis of minimal risk dose escalation, 3 different 4π planning schemes, including dose escalation or margin increase, were investigated.

As suggested by the literature, dose escalation from 60 Gy to 100 Gy or greater is needed to significantly improve GBM patient survival. In the first two schemes, we prescribed 100 Gy to cover 95% of the PTV and GTV, named 4πPTV_100Gy_ and 4πGTV_100Gy_, respectively. In the 4πGTV_100Gy_ scheme, the remaining PTV was covered by the prescription dose. Literature also suggests that in addition to the high central recurrence rates, GBM is also likely to recur at the field margin. In the last scheme, the original PTV was further expanded by 5 mm while maintaining the original prescription doses (4πPTV_PD+5mm_). The expanded margins were adjusted to not extend beyond the outer skull and to avoid critical structures, unless the original PTV already encompassed part of the structure. Doses to the normal brain, which excluded the PTV contour, were evaluated. For the 4πPTV_PD+5mm_ plan, the normal brain was still defined to exclude the original PTV.

The Wilcoxon signed-rank test was utilized to compare the 4π plans against the clinical plan. Evaluated OARs included the brain, brainstem, chiasm, spinal cord, eyes, lenses, optical nerves, and cochleae.

## Results

**Figure 1:**
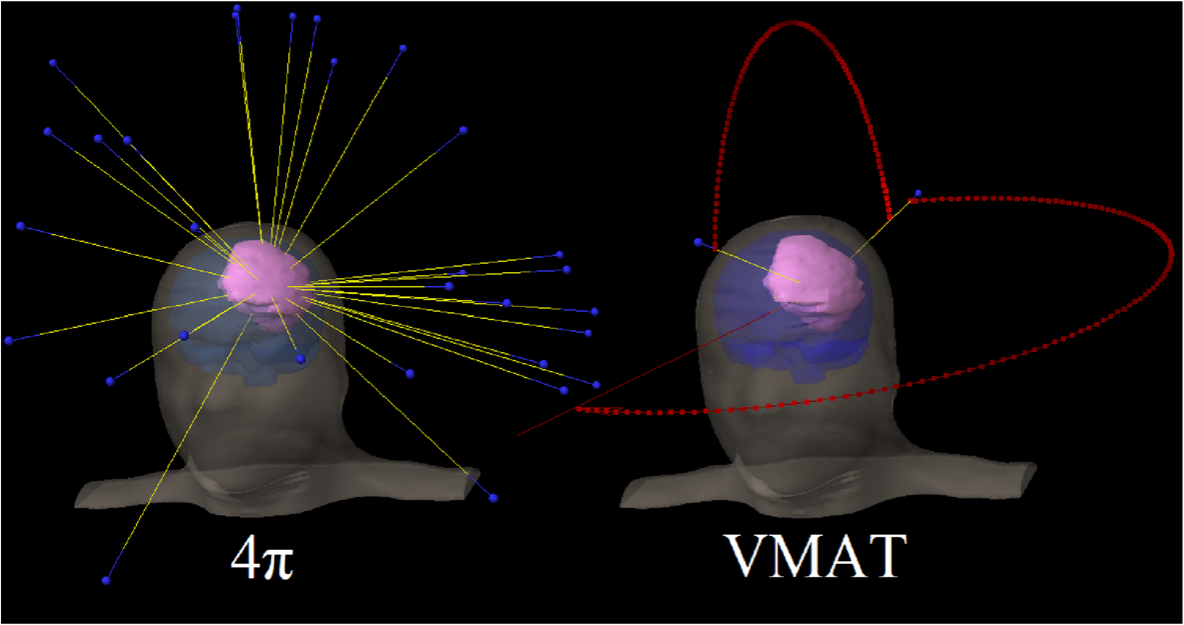
**Typical beam patterns of 4π beams vs. VMAT beams.**

Figure 1 shows the beam entrance patterns of a typical non-coplanar 4π plan and the corresponding VMAT plan utilizing two partial non-coplanar arcs.

All of the 4π plans exhibited a decrease in maximum and mean doses for the OARs exclusive of the brain. Table 2 lists the average percent maximum and mean OARs dose *reductions* for 4π compared to the clinical plans. The 4πPTV_PD_ plans significantly reduced (p < =0.003) the maximum and mean brainstem dose by 47% and 61%, respectively, and reduced (p < = 0.007) doses to all other non-brain OARs by a range of 74-98% and 90-99%, respectively. The 4πPTV_100Gy_ plans produced unchanged (p > 0.05) maximum and mean doses in the brainstem, and a significant decrease (p < = 0.005) in the maximum and mean doses to other OARs, exclusive of the brain, in the range of 65-95% and 78-97%, respectively, except for an insignificant change (p > 0.05) in the spinal cord dose when compared to the clinical plans. The 4πGTV_100Gy_ study did not change the brainstem maximal doses (p > 0.05), but reduced its mean doses by 57.77% (p < 0.001). The remaining OARs, exclusive of the brain, showed a significant decrease (p < 0.006) in the maximum and mean doses by a range of 84-99% and 86-99%, respectively. The 4πPTV_PD+5mm_ plan significantly reduced the (p < = 0.003) maximum and mean doses for the brainstem by 31% and 52%, respectively, and reduced (p < 0.04) the maximum and mean doses to other OAR, exclusive of the brain, by a range of 67-98% and 60-99%, respectively, except for the unchanged (p > 0.05) maximum doses in the spinal cord and left cochlea, when compared to the clinical plans.

**Table 2:** **The average maximum and mean dose reduction (%) of the OARs except the normal brain**

**Normal tissue comparison**	**4πPTV**_**PD**_		**4πPTV**_**100Gy**_		**4πGTV**_**100Gy**_		**4πPTV**_**PD+5mm**_	
	**Average Max Dose Reduction (%)**							
	**%**	**p-value**	**%**	**p-value**	**%**	**p-value**	**%**	**p-value**
Brainstem	47.01	0.003	7.18	0.898	27.65	0.123	30.66	0.003
Chiasm	84.73	<0.001	72.12	<0.001	86.74	<0.001	77.06	<0.001
Spinal Cord	73.98	0.007	39.79	0.240	84.22	0.006	51.94	0.064
Left Eye	83.89	<0.001	69.03	<0.001	87.03	<0.001	66.86	<0.001
Right Eye	88.01	<0.001	76.16	<0.001	88.11	<0.001	84.12	<0.001
Left Lens	98.43	<0.001	94.96	<0.001	98.63	<0.001	97.53	<0.001
Right Lens	97.74	<0.001	93.88	<0.001	98.82	<0.001	97.59	<0.001
L Opt Nrv	87.12	<0.001	74.31	<0.001	88.45	0.002	72.44	<0.001
R Opt Nrv	89.93	<0.001	80.42	<0.001	91.33	0.005	75.72	<0.001
L Cochlea	82.45	<0.001	64.87	0.005	84.15	0.003	44.94	0.067
R Cochlea	93.58	<0.001	85.19	0.001	93.10	<0.001	77.97	0.007
	Average Mean Dose Reduction (%)							
Brainstem	60.96	0.002	38.08	0.067	57.77	<0.001	51.87	0.002
Chiasm	95.30	<0.001	88.88	<0.001	96.23	<0.001	92.27	<0.001
Spinal Cord	92.43	<0.001	80.07	<0.001	98.08	<0.001	71.51	0.014
Left Eye	94.61	<0.001	85.66	<0.001	96.49	<0.001	89.78	<0.001
Right Eye	95.94	<0.001	87.95	<0.001	96.69	<0.001	94.24	<0.001
Left Lens	99.23	<0.001	97.11	<0.001	99.44	<0.001	98.56	<0.001
Right Lens	99.10	<0.001	96.56	<0.001	99.47	<0.001	98.72	<0.001
L Opt Nrv	95.98	<0.001	87.66	<0.001	96.96	<0.001	88.46	<0.001
R Opt Nrv	97.48	<0.001	94.08	<0.001	98.42	<0.001	93.09	<0.001
L Cochlea	90.34	<0.001	78.41	0.002	85.92	0.002	60.16	0.037
R Cochlea	96.83	<0.001	92.81	0.003	97.79	<0.001	90.83	0.002

**Figure 2:**
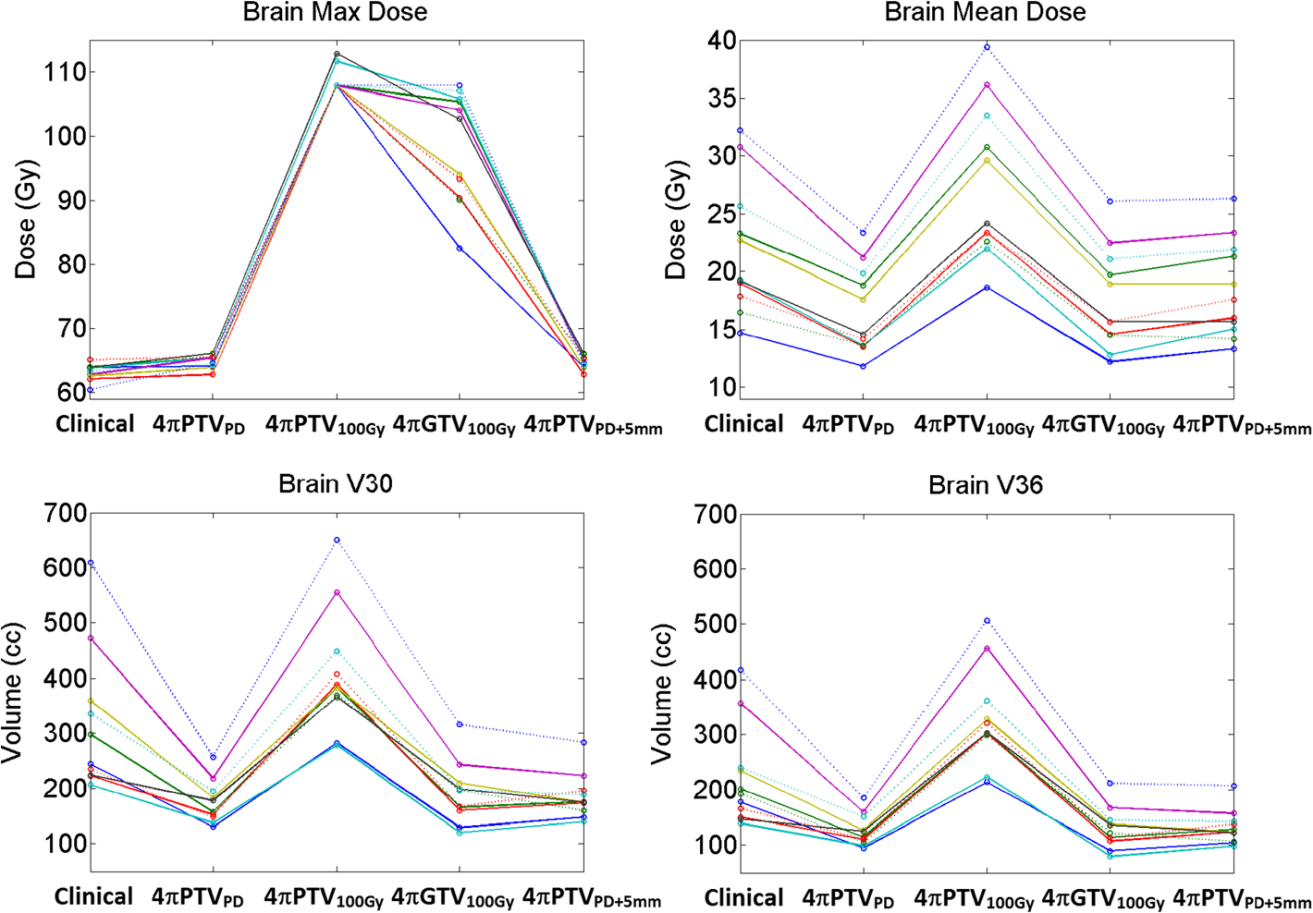
**Individual patient dosimetry comparisons between clinical VMAT plans and the various 4π plans.**

Figure 2 compares the normal brain maximum dose, mean dose, V30, and V36 between the clinical plan and the 4π plans. The 4πPTV_PD_ plans significantly reduced (p < 0.001) the mean brain dose from 21.94 Gy to 16.56 Gy and slightly increased the maximum normal brain doses (p < 0.001) from 63.24 to 64.88 Gy. The maximum normal brain doses increased (p < 0.001) in the 4πPTV_100Gy_ and 4πGTV_100Gy_ plans to 108.71 Gy and 98.51 Gy respectively. The 4πPTV_100Gy_ plans also significantly increased (p < 0.001) the mean brain dose to 27.60 Gy (26%), while the 4πGTV_100Gy_ significantly decreased (p < 0.001) the mean brain dose to 17.61 Gy (20%). For the 4πPTV_PD+5mm_ plans, the maximum brain dose slightly increased (p < 0.001) from 63.24 Gy to 64.94 Gy, and the brain mean dose was statistically unchanged (p > 0.05).

**Figure 3:**
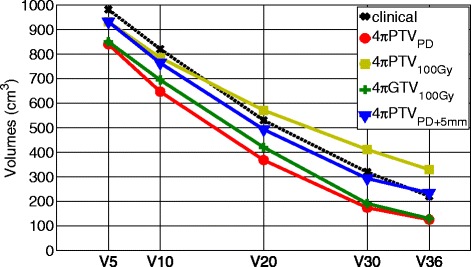
**Comparison of volumes between the clinical and 4π plans.**

Brain dose comparison for individual patients is shown in Figure 2. V36 and V30, surrogates for potential brain necrosis, were significantly decreased (p < 0.001), for the 4πPTV_PD_ and the 4πGTV_100Gy_ plans, statistically unchanged for the 4πPTV_PD+5mm_ plan, and significantly increased (p < 0.001) for the 4πPTV_100Gy_ plan. In the 4πPTV_PD_ and 4πGTV_100Gy_ plans, the brain V36 significantly decreased (p < 0.001) by 94.77 cm^3^, 90.92 cm^3^, respectively. Similarly, the brain V30 decreased by 144.92 cm^3^, 127.63 cm^3^ for the same plans. The brain V36 and V30 of the 4πPTV_100Gy_ plan significantly increased (p < 0.001) by 108.62 cm^3^ and 91.81 cm^3^, respectively. V5, V10, and V20 were also significantly decreased (p < 0.01) in the the 4πPTV_PD_ and 4πGTV_100Gy_ plans, and statistically unchanged (p > 0.05) in the 4πPTV_PD+5mm_ and 4πPTV_100Gy_ plan. A comparison of the average volumes plotted against the various “Vx” are shown in Figure 3.

**Figure 4:**
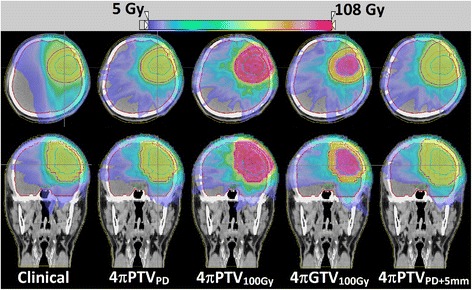
**Dose wash of the clinical case and the various 4π plans for a single patient.**

**Figure 5:**
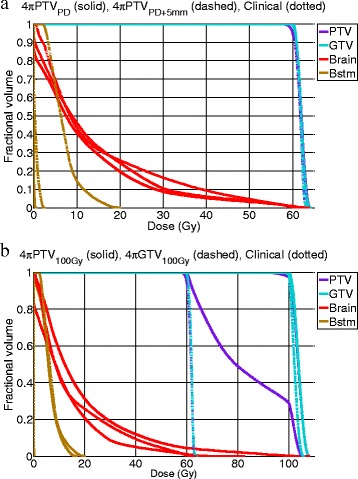
**Cumulative dose volume histogram comparisons for a typical patient. (a)** Dose volume histogram comparing the clinical plan to the 4πPTV_PD_ plan and the 4πPTV_PD+5mm_ plan. **(b)** Dose volume histogram comparing the clinical plan to the 4πPTV_100Gy_ plan and the 4πGTV_100Gy_ plan.

Figure 4 shows a dose colorwash superimposed on the planning CT for a typical case. The dose fall-off outside the PTV is steeper in the 4π cases. R50 was significantly lowered from 2.52 in the clinical plans to 1.97 (4πPTV_PD_), 2.00 (4πPTV_100Gy_), 2.10 (4πGTV_100Gy_), and 1.75 (4πPTV_PD+5mm_) respectively. The DVHs shown in Figure 5 compare the PTV and the brain doses. With the exception of the 4πPTV_100Gy_ plans, the 4π plans reduced dose to the brain and brainstem while maintaining superior doses to the tumor. 4π plans resulted in essentially equivalent or slightly superior PTV coverage compared to the clinical plans.

## Discussion

GBM tumors are nested within the normal brain tissue and are often proximal to other serial critical organs. Currently established dose of approximately 60 Gy to the GBM tumor has shown survival benefit compared to lower doses but milder dose escalation from this dose has not improved patient survival or local control. Dose escalation studies to over 100 Gy or therapy using boron neutron capture therapy [19] with high radiobiological equivalent dose show that very aggressive dose escalation could provide meaningful improvement in tumor response. However, attempting dose escalation without drastically improving the dose compactness will result in a greater severe toxicity risk long before reaching a tumor doses that can significantly delay GBM recurrence, let alone the dose needed for permanent local tumor control. Previous dose escalation studies utilizing 3D conformal radiation therapy have shown worse patient survival [4,5] largely due to the lack of dose conformity around the target. Compared to 3D conformal radiation therapy used in the aforementioned clinical trials, intensity modulated radiation therapy (IMRT) better conforms radiation doses to the target. However, intensity modulation does not fundamentally change the “compactness” of dose distribution [20], which has been commonly defined using the ratio of the 50% isodose volume to the PTV volume. Previous dose escalation studies to beyond 100 Gy BED had to utilize proton therapy or brachytherapy, severely limiting their applicability [8-10]. Brachytherapy particularly increases the bleeding and infection risk, making it a less appealing option for many clinics.

It has been previously shown that 4π radiotherapy will provide improved dose compactness compared to coplanar arc and manually selected non-coplanar IMRT approaches [12]. Selecting and optimizing beams in the vast non-coplanar solution space is an enormously complex problem that has become manageable using innovative optimization algorithms such as the column generation approach. Using our 4π research-planning platform, we showed that GTV dose escalation to 100 Gy is achievable while maintaining lower mean and maximum OAR doses. Use of this technique may result in delayed or reduced central recurrences. To effectively reduce marginal recurrences, dose escalation to the entire PTV or more is needed. We showed that by using 4π, an additional 5 mm can be added without increasing normal tissue doses. The 4πPTV_100Gy_ plans unavoidably increased the brain maximum and mean doses and V30 and V36. It remains to be seen whether these doses could be safely delivered, but if a consistent fractionation is employed, the greater doses will be delivered using more fractions. For example, delivering the 100 Gy in 50 fractions instead of the 30–33 fractions required for the original plan, the biological equivalent dose to normal brain tissue would be decreased from between 211.11 and 201.01 Gy to 166.67 Gy.

Like all dosimetry comparison studies, there is often question whether the best possible plan has been achieved in either planning platform. While subjective biases cannot be completely ruled out, the large differences in normal organ doses exceed typical variation in plan quality from the same planning platform. Furthermore, R50 as a measurement of dose compactness has been a highly reproducible parameter that is not substantially affected by the selection of penalties and constraints in optimization. Therefore, the observed significant difference in R50 is unlikely due to operators.

Therefore, with the advent of 4π radiotherapy, it is now feasible to employ markedly improved dose conformity and compactness for meaningful GBM dose escalation using external beam X-rays alone. 4π radiotherapy using C-arm gantries is less invasive than brachytherapy and more accessible than proton beams. There are practical hurdles to deliver these plans, namely collision avoidance and delivery efficiency. While collision angles can be excluded based on the patient specific surface measurements, the delivery time will include the greater time required to move the couch and gantry between the numerous beams. Based on our experience delivering 4π test plans on a TrueBeam machine (Varian, Palo Alto) using automated sequencing, the total time for the machine to travel between 30 non-coplanar beams was less than 200 seconds and total treatment time from the first to the last beam less than 15 minutes. The couch rotation in a typical 4π treatment involves many small steps totaling less than 180°. Such magnitudes of couch kicks are routinely used in linac based brain stereotactic radiosurgery and appear to be well tolerated. Overcoming these practical hurdles and integrating 4π into a clinical delivery flow will allow for equivalent or better patient survival than the previous dose escalation studies.

## Conclusion

The significantly improved dose coverage and high dose conformity of 4π radiotherapy made it possible to escalate the GBM prescription doses from 60 Gy to 100 Gy or to increase the PTV margins while meeting clinical critical organ dose constraints. For GBM, this 4π non-coplanar radiotherapy modality may subsequently improve the GBM treatment outcome.

### Consent

An institutional review board (IRB) protocol has been approved for retrospectively accessing and the publication of anonymized patient data. Individual patient consent was not necessary.
